# Primary Sarcoma of Descending Aorta

**DOI:** 10.1055/s-0039-3401809

**Published:** 2020-04-09

**Authors:** Adele Tessitore, Alessio V. Mariolo, Domenico Galetta, Giulia Sedda, Rosa Spirito, Lorenzo Spaggiari

**Affiliations:** 1 Department of Thoracic Surgery, IEO, European Institute of Oncology IRCSS, Milan, Italy; 2 Department of Cardiovascular Surgery of the University of Milan, Centro Cardiologico Monzino IRCSS, Milan, Italy; 3 Department of Oncology and Hemato-Oncology, University of Milan, Milan, Italy

**Keywords:** intimal sarcoma, aorta reconstruction, descending aorta

## Abstract

Primitive aortic sarcomas are rare tumors characterized by resistance to medical treatment and a poor prognosis with high metastatic rates and local recurrences. Surgery remains the mainstay treatment and is based on challenging and technically demanding resections with high rate of major intraoperative and postoperative complications. We report the case of a patient with primitive intimal sarcoma of the aorta, who underwent a descending aortic resection and reconstruction with a prosthetic tube.

## Introduction

Intimal sarcomas are extremely rare tumors involving the great vessels, primarily the pulmonary artery and the aorta, with an unfavorable prognosis. 1 To date, the role of neoadjuvant chemotherapy is debated and surgery still remains the mainstay option when the tumor is resectable. 2

We report the case of a surgically treated primary sarcoma of the aorta.

## Case Presentation

A 48-year-old male with a history of chronic cardiac disease was referred to our division for 3 months of persistent back pain.

**Fig. 1:**
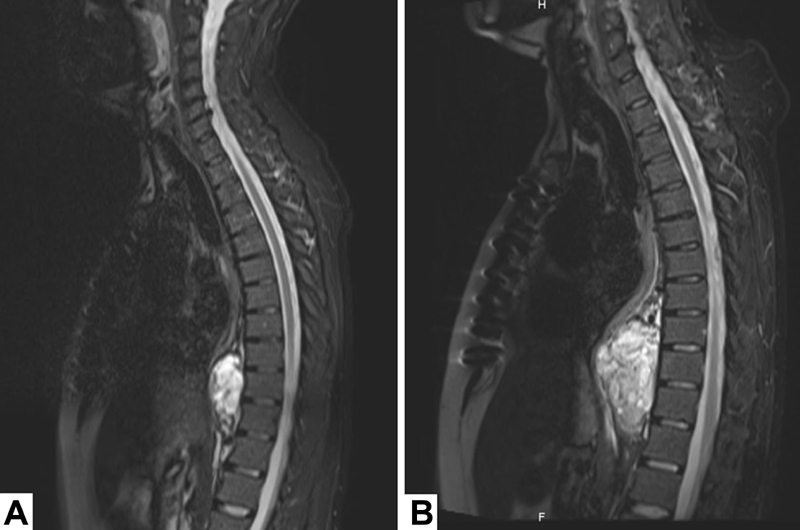
Magnetic resonance imaging ( **A** ) at the diagnosis and ( **B** ) after post induction chemotherapy.

A computer tomography (CT) scan and a magnetic resonance imaging (MRI, Fig. 1A ) revealed a solid fusiform lesion of the descending aorta, with no free plane and suspected parietal infiltration. The lesion had axial diameters of 35 mm × 34 mm and a longitudinal extension of 56 mm, with an inhomogeneous structure, peripheral contrast enhancement and intralesional septa. 18-F–fluorodeoxyglucose positron emission tomography (18F–FDG PET) was performed, revealing isolated uptake by the lesion without evidence of distant metastasis.

To obtain a histological diagnosis of the tumor, a CT-guided biopsy was attempted without success. The diagnosis was later achieved with a left thoracoscopic surgical biopsy and the final histopathology revealed a primitive sarcoma of the aortic intima.

The patient underwent neoadjuvant chemotherapy treatment with one cycle of epirubicin and taxolo. A new MRI ( Fig. 1B ) revealed progression of the tumor.

Considering the patient characteristics, the clinical investigations, and the poor response to chemotherapy, a multidisciplinary discussion suggested a salvage surgical resection.

**Fig. 2:**
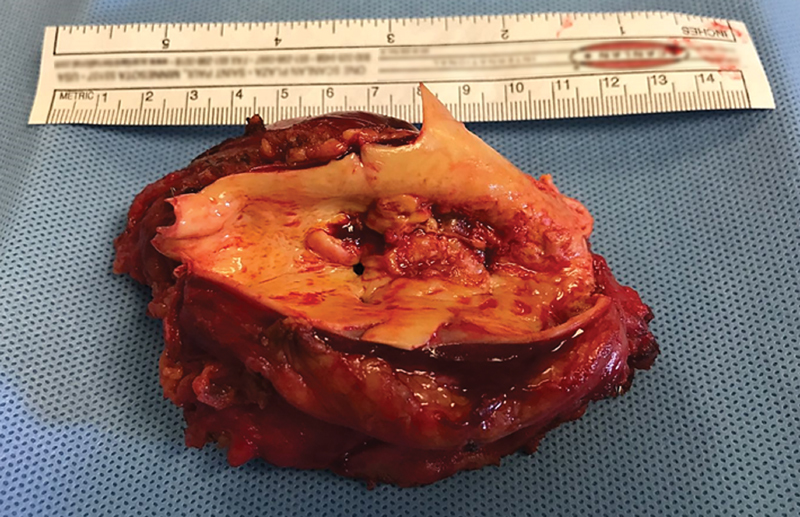
Surgical specimen.

A left thoracoabdominal approach was chosen. Under full heparinization, the descending thoracic aorta was isolated, clamped, resected, and reconstructed with a prosthetic tube of Dacron (18 mm; Video 1 ), positioned with an end-to-side anastomosis above the celiac trunk before that the complete resection of the neoplasm was performed ( Fig. 2 ).

**Video 1**

Aorta reconstruction with a prosthetic tube of Dacron.

Histopathological analysis confirmed the diagnosis of intimal sarcoma with greater than 50% necrosis, pleomorphism, and a mitotic index exceeding to 10 × 10 power fields. The immunohistochemical staining was positive for ETS-related gene protein and ubiquitin carboxyterminal hydrolase and focally positive for CD 31, factor VIII, and S100. The intimal layers of the resected aorta were involved with margins microscopically free.

At the end of surgery, the patient showed flaccid paraplegia with loss of anal sphincter control and a tactile hypoesthesia. These signs were associated with a spinal cord injury at the L1/L2 level. The patient was discharged on the 10th postoperative day. However, he was readmitted due to dyspnea after 2 days. CT-scan revealed a right iliac venous thrombosis extending to the femoral vein, requiring an urgent inferior vena cava filter placement.

After 2 months of follow-up another MRI showed a local recurrence and liver metastases, for which the patient started an immunotherapy treatment with pazopanib 800 mg/day. The local recurrence and the hepatic metastases were reduced after 2 months of treatment and metabolically silent on 18-F–FDG PET imaging.

After 1 year, the patient is still alive with persistent paraplegia for which he has started an intensive neurological rehabilitation program.

## Discussion

Primary sarcomas of the aorta are extremely rare 1 and are usually located in the descending thoracic aorta. Clinically, the symptomatology is nonspecific; patients often present with systemic signs of tumor fragment embolism, such as limb claudication or mesenteric ischemia. Rarely, the tumor can be revealed by the rupture of an aneurysm often misdiagnosed as atherosclerotic. These confounding symptoms explain the frequent delayed diagnosis, usually achieved only when metastatic peripheral emboli become symptomatic. 3 Thus, the preoperative diagnosis of aortic sarcoma is challenging, as in our case. CT or MRI imaging may be helpful to differentiate aortic sarcomas from other aortic disease.

The prognosis of aortic sarcomas is very poor, related to the tumor histological grade, possibility of resection and anatomic location. The average survival rate is 15.6 months after diagnosis. 4

Although several measures have been developed to reduce the incidence of spinal ischemia and neurological complications, this type of surgery is still associated with a high rate of postoperative complications, with up to 16% paraplegia. 5

Adjuvant chemotherapy with doxorubicin is indicated in case of tumor emboli, metastases, or unresectable tumors, with response rates of 10 to 25%. Other proposed medical treatments include ifosfamide, gemcitabine, or docetaxel. The use of the immunotherapy shows promising success and worthy of further evaluation.

In conclusion, the optimal treatment for aortic angiosarcoma is still under investigation and may be considered a multimodal treatment. Surgery mainly represents a salvage option and is particularly indicated in young patients to achieve a radical resection even if the postoperative complication rates remain high.
